# Immunomic Investigation of Holocyclotoxins to Produce the First Protective Anti-Venom Vaccine Against the Australian Paralysis Tick, *Ixodes holocyclus*

**DOI:** 10.3389/fimmu.2021.744795

**Published:** 2021-10-04

**Authors:** Manuel Rodriguez-Valle, Sonia McAlister, Paula M. Moolhuijzen, Mitchell Booth, Kim Agnew, Claudia Ellenberger, Aleta G. Knowles, Kathleen Vanhoff, Matthew I. Bellgard, Ala E. Tabor

**Affiliations:** ^1^ Centre for Animal Science, The University of Queensland, Queensland Alliance for Agriculture & Food Innovation, St. Lucia, QLD, Australia; ^2^ Centre for Comparative Genomics, Murdoch University, Murdoch, WA, Australia; ^3^ Paul Dick & Associates Ltd, Castle Hill, NSW, Australia; ^4^ Elanco Animal Health, Yarrandoo Research and Development Centre, Kemps Creek, NSW, Australia; ^5^ Virbac Australia Pty Ltd, Milperra, NSW, Australia; ^6^ eResearch Office, Queensland University of Technology, Brisbane, QLD, Australia; ^7^ School of Chemistry and Molecular Biosciences, The University of Queensland, St. Lucia, QLD, Australia

**Keywords:** cocktail vaccine, anti-paralysis vaccine, paralysis tick, *Ixodes holocyclus*, vaccine

## Abstract

Venom producing animals are ubiquitously disseminated among vertebrates and invertebrates such as fish, snakes, scorpions, spiders, and ticks. Of the ~890 tick species worldwide, 27 have been confirmed to cause paralysis in mammalian hosts. The Australian paralysis tick (*Ixodes holocyclus*) is the most potent paralyzing tick species known. It is an indigenous three host tick species that secretes potent neurotoxins known as holocyclotoxins (HTs). Holocyclotoxins cause a severe and harmful toxicosis leading to a rapid flaccid paralysis which can result in death of susceptible hosts such as dogs. Antivenins are generally polyclonal antibody treatments developed in sheep, horses or camels to administer following bites from venomous creatures. Currently, the methods to prevent or treat tick paralysis relies upon chemical acaricide preventative treatments or prompt removal of all ticks attached to the host followed by the administration of a commercial tick-antiserum (TAS) respectively. However, these methods have several drawbacks such as poor efficacies, non-standardized dosages, adverse effects and are expensive to administer. Recently the *I. holocyclus* tick transcriptome from salivary glands and viscera reported a large family of 19 holocyclotoxins at 38-99% peptide sequence identities. A pilot trial demonstrated that correct folding of holocyclotoxins is needed to induce protection from paralysis. The immunogenicity of the holocyclotoxins were measured using commercial tick antiserum selecting HT2, HT4, HT8 and HT11 for inclusion into the novel cocktail vaccine. A further 4 HTs (HT1, HT12, HT14 and HT17) were added to the cocktail vaccine to ensure that the sequence variation among the HT protein family was encompassed in the formulation. A second trial comparing the cocktail of 8 HTs to a placebo group demonstrated complete protection from tick challenge. Here we report the first successful anti-venom vaccine protecting dogs from tick paralysis.

## 1. Introduction

Venom producing animals are ubiquitously distributed among vertebrates and invertebrates like fish, snakes, scorpions, spiders, and ticks (1, 2). Snake venoms are amongst the most highly characterized of animal venoms and are conformed by a complex mixture of pharmacologically active proteins and peptides (3) conferring their toxicological property. Due to its incidence and human impact, the World Health Organization recently recognized snakebite as a neglected tropical disease that affects ∼ 2.7 million per annum (3).

However, hematophagous invertebrates such as ticks are not as well recognized as venomous animals (1). There are approximately 890 tick species worldwide with 73 species confirmed to be associated with host paralysis (4). These include 27 species with evidence of paralysis, such as soft tick species (n=8) from the genera *Argas* and *Ornithodoros* which paralyze mostly livestock, and also hard tick species (n=19) from the genera *Ixodes*, *Amblyomma*, *Dermacentor*, *Haemaphysalis*, *Hyalomma* and *Rhipicephalus* which paralyze a broader list of hosts including humans, livestock, reptiles, companion animals and rabbits (1, 4, 5). *Ixodes holocyclus* and *Ixodes cornuatus* cause paralysis in humans, domestic animals, and wildlife with an eastern coast and southern Australian distribution respectively (6). The Australian paralysis tick (*I. holocyclus*) is the most important tick species associated with paralysis in Australia distributed along the eastern coast from North Queensland to the Lakes Entrance in the southern state of Victoria (6). It has been reported from an unpublished survey to cause tick paralysis in approximately 10,000 to 100,000 animals annually with a death rate of around 5% (7). The toxicity of *I. holocyclus* is caused by a family of neurotoxins named ‘holocyclotoxins’ present in the tick saliva. The only toxin molecules that have been characterized for any tick species is for the Australian paralysis tick (8, 9), including demonstrated specific paralysis activity in the mouse neonate model (8, 10). Additionally, there are several bacterial pathogens transmitted by Australian ticks such as *I. holocyclus* including *Rickettsia australis* (Queensland tick typhus) and *Coxiella burnetii* (Q fever). Recently, *I. holocyclus* tick bites have been confirmed as the trigger for a mammalian meat allergy in humans (11). Despite evidence from scientific studies (transcriptomic, sentinel studies and antibody screens) failing to show that the causative agent of Lyme disease *Borrelia burgdorferi* sensu lato is present in Australia (10, 12), a similar multisystem disorder has been identified with similar symptoms in humans (13). Currently, in Australia, there is no formal reporting system of paralysis tick cases with incidence data only available from focused geographical or specific annual survey reports (14, 15).

Antivenoms based on polyclonal antibodies for treatments against venoms of ticks, snakes, spiders, scorpions and other species are produced in animals such as dogs, horses, camels and sheep with research focusing on identifying safer inoculation schedules (16–20). These antivenoms are the most effective method to abrogate and treat paralysis by reducing the effect of postsynaptic neurotoxins (21). Ticks are much smaller producing minute volumes of saliva and thus collecting venom, or salivary gland secretions requires a large number of ticks. For ticks, where their size precludes physical extraction of venom, antivenoms are produced commercially by feeding ticks on canine hosts to obtain hyper-immune tick anti-serum (22, 23). The antivenoms applied for presynaptic neurotoxins (such as those present in the South American rattlesnake and the Australian paralysis tick) is less successful if the paralysis has progressed prior to treatment (24, 25). The paralysis caused by these species are considered incidental and as such vaccines do not appear to be a priority as the delivery of treatment for bitten individuals deemed the best approach to control neurotoxic consequences (26, 27).

Chemical acaricides administrated topically or *via* collars have been the most common methods used to prevent and control paralysis ticks on companion animals (28). Oral acaricides based on isoxazoline chemicals sold as Bravecto™ and NexGard^®^ have been introduced (29, 30) which aim to control both paralysis ticks and fleas. New long acting collars based on slow release Imidacloprid/flumethrin (Seresto^®^) have shown promise more recently (31). However, the risk of ticks developing resistance remains and the adverse reactions of these drugs warrant the treatments unsafe for some dogs and as such new drugs continue to be developed by companion animal product companies (32, 33). These preventative methods have drawbacks with efficacies of less than 100%, and a single surviving tick(s) can kill a large dog (34), however the risk is higher for smaller dogs as neurotoxin dose correlates with host weight (14). Cats are more sensitive to these drugs thus other options have been developed using dog orals as topicals with a recent study examining 2077 cat cases to conclude that the mortality risk for cats is low from paralysis caused by *I. holocyclus* (35). The current treatment for tick paralysis relies on the prompt removal of all attached ticks and the administration of a commercial tick antiserum (TAS). Morbidity and mortality rates decrease with TAS introduction. It also has many drawbacks including: inhumane production (*via* dog hyper-immunization), limited window of utility (needing to be administered in the early stages of disease), non-standardized dosage, side effects and varying potency between batches and manufacturers (reviewed by 26). In the last few years, commercial production of TAS has reduced to only one Australian company (36), suggesting that the use of new oral drugs in dogs has decreased the number for dog paralysis cases and also the TAS commercial production need.

The notion of an anti-paralysis tick vaccine and the protective attributes of hyperimmune dog serum to treat cases of paralysis were first developed more than 80 years ago in a controlled study with paralysis tick infected dogs (22, 23). Later studies also demonstrated tick infestation of dogs to produce hyperimmune dog serum (37), or to demonstrate protection from tick challenge, which correlated to serum anti-toxin antibody titers (38). Other studies using whole tick homogenates (39) or dissected salivary gland extracts ‘toxoid’ also demonstrated protection from challenge with *I. holocyclus* in controlled dog studies. These studies required large numbers of ticks and the preparation of native protein for the production of a potential crude vaccine which was not commercially feasible. It was not until the 1990s that neurotoxins or holocyclotoxins bound to rat brain synaptosomes (pinched-off nerve terminals) were isolated as three polypeptides HT1, HT2 and HT39. Following N-terminal sequencing, a partial sequence of holocyclotoxin 1 (HT1) was obtained, and PCR technologies were utilized to obtain the gene sequence (40). It was not until 2014 and 2018 that the structure of a chemically synthesized HT1 demonstrated four disulphide bonds with three contributing to inhibitory cysteine knot motif, and transcriptome sequencing identified a large holocyclotoxin family of up to 19 neurotoxins respectively (10, 41). The holocyclotoxin sequence identities varied from 38-99%, suggesting the development of a vaccine may be challenging.

Currently, there are 19 characterized HTs (10), and identifying the holocyclotoxins critical to paralysis may assist future vaccine development. The present study aims to identify immunogenic HTs following screening with dog anti-paralysis tick serum. Here we describe the first successful anti-venom vaccine demonstrating the protection of dogs from *Ixodes holocyclus* challenge.

## 2. Materials and Methods

### 2.1 Holocyclotoxins and Immunogenicity Screening With Polyclonal Tick Anti-Serum

A transcriptome database was produced following cDNA sequencing of organs dissected from *I. holocyclus* females collected from paralyzed dogs and cats as previously described (10). Subsequently to the publication in 2019, the database was deposited into Genbank as the transcriptome shotgun assembly (TSA) under accession GIBQ00000000 (see ‘Data Availability Statement’ for more details). Transcriptome sequence data was mined for homologues of HT1 (Accession AAV34602) and an additional 18 full length transcripts were described with amino acid sequences under accessions: HT2 (KP096302), HT3 KP096303), HT4 (KP963966), HT5 (KP096304), HT6 (KP096305), HT7 (KP096306), HT8 (KP096307), HT9 KP096308), HT10 (KP096309), HT11 (KP096310), HT12 (KP963967), HT13 (KP963968), HT14 (KP963969), HT15 (KP963970), HT16 (KT439073), HT17 (KT439074), HT18 (KT439075), and HT19 (KT439076), respectively. All HTs used in ELISAs and immunizations were synthesized as previously described using Fmoc chemistry (10).

Three different Summerland purified anti-tick serum batches (2012-2014) (42) containing purified dog anti-tick immunoglobulins at 500 anti-toxin units (ATU) per bottle were screened against 19 synthetic holocyclotoxins produced as previously described (10) using ELISA. Nunc-Immuno™ MicroWell™ 96 well plates (Sigma-Aldrich, Australia) were coated with 100 ng/well of each synthetic holocyclotoxin diluted in carbonate buffer (0.1M sodium carbonate-bicarbonate solution, pH 9.6) in duplicate and incubated overnight at 4°C. HT13 was not screened due to poor synthetic peptide preparation (data not shown). Control wells were coated with 100 ng of *I. holocyclus* salivary gland extract (positive control) or bovine serum albumin (BSA) (negative control). Plates were washed three times with 200 μL wash buffer (WB: 0.05% Tween 20 in 10mM phosphate buffer saline, pH 7.4) before blocking the wells with 200 μL blocking buffer (BB: Pierce™ Protein Free PBS Blocking Buffer, Thermo Scientific, Australia) overnight at 4°C. After blocking, the plates were washed three times with 200 μL WB. Serial dilutions of Summerland TAS starting at 1:500 in BB were added across each row of the plates as the primary antibody and incubated for 1hr at room temperature (RT) on a platform shaker (Ratek Instruments, Australia). After three washes, 100 μL of horseradish peroxidase (HRP) conjugated- Sheep Anti-Dog IgG (abcam^®^, Australia) diluted 1: 10,000 in BB were added to the plates and incubated for 30 minutes at room temperature. Subsequently, plates were washed five times with WB before 100 μL of 3,3’,5,5’- Tetramethylbenzidine (TMB) was added to each well (KPL, USA) and allowed to develop for 10 minutes. The reaction was stopped with 100 μL 1M phosphoric acid. Absorbance was measured using the BioTek Epoch Spectrophotometer with λ= 450nm filter (Millennium Science, Australia) and the end point titer was calculated as the reciprocal of the dilution that reached the background absorbance of the negative control (OD λ= 450nm).

### 2.2 Dog Challenge Trials

These studies were conducted under the Australian Pesticide and Veterinary Medicine Authority (APVMA) Small-scale Trials Permit PER7250 at an R & D Centre, New South Wales, Australia, approved by the Elanco Animal Ethics Committee. Peptides for the dog trials were synthesized as previously described using fmoc chemistry and confirmation of peptide folding (10, 41).

#### 2.2.1 Trial 1

An exploratory immunization experiment was carried out to determine if HT peptide structure was important for immunogenicity using three animal groups consisting of one adult dog per group. The ‘unfolded’ preparation contained 1mM Dithiothreitol (DTT) to reduce the disulphide bonds forming between cysteine residues of the HT peptides. Three adult female kelpie cross Labrador dogs from the same litter of 5.25 years of age at 18.9-20.2kg in weight were randomized into three treatment groups. Group II (Dog #62469) and III (Dog #63458) were immunized with unfolded and folded peptides respectively, and Group I was the placebo control (Dog #70084) treated with PBS and Incomplete Freund’s adjuvant (IFA). The immunization was subcutaneous in the neck with 1 mL of IFA containing 60 µg (~10.83 nmol) each of 5 HTs (HT1, HT2, HT3, HT4 and HT12) – thus a total of 300 µg (54.15 nmol) of protein). The first immunization was Day 0 with two booster injections at 14 and 28 days after the first dose. The dogs were observed for signs of tick paralysis as summarized in **Supplementary Table 1**.

#### 2.2.2 Trial 2 

A double blinded, placebo-controlled pilot study designed to compare the efficacy and safety of the peptide cocktail vaccine and determine the antibody responses. The dog cohort consisted of three Beagles, one Beagle cross, two Huntaways, one Huntaway cross and one Kelpie cross as two de-sexed males, four de-sexed females and two fertile females. The dogs were two years and six months old with one dog eight years old. The eight dogs were randomized into two groups and treated with either the vaccine (HT cocktail, Group 2, Dog IDs: 34295, 55891, 64799, 68888; #55891 included the single 8-year-old dog) or a placebo control (adjuvant only, Group 1, Dog IDs: 62458, 63061, 66324, 99768). Eight HT peptides (HT1, HT2, HT4, HT8, HT11, HT12, HT14 and HT17) were formulated in IFA at 30μg (5.42 nmol) per peptide with a total of 240μg (43.32 nmol) per dose per dog. These were administered in three subcutaneous doses on days 0, 28 and 49. The dogs were observed for signs of tick paralysis as determined by clinical observation and evaluated against a matrix of clinical and subclinical signs, as summarized in **Supplementary Table 1**.

### 2.3 Sera Collection

Sera were collected on days 0 (baseline), and 14, 28, 42 and 50 (Trial 1), and 28, 42, 63 and 66 (Trial 2) with antibody levels to the HTs determined by ELISA. Each dose was delivered to a new site (left and right shoulder) to allow for the monitoring of injection site reactions. After each vaccination the dogs were observed for signs of tick toxicosis every 3 hours ( ± 30 mins) for a period of 12 hours post vaccination. Personnel evaluating the dogs were blinded.

### 2.4 Tick Challenge

The ticks were sourced from the Tweed Heads region of Northern New South Wales, Australia (Latitude 28.1787° S, Longitude 153.5370° E). Two weeks after the third vaccination (trial 1 day 42, trial 2 day 63), in both trials the dogs were challenged with one unfed adult female *I. holocyclus* ticks to induce symptoms of toxicosis. At three days after tick attachment (trial 1 day 45, trial 2 day 66, 72h post tick attachment) the dogs were observed every 3 hours ( ± 30 mins) for signs of toxicity until the study end (trial 1 day 50, trial 2 day 70). Signs of tick toxicosis were determined by clinical observation performed by an appropriately trained person and evaluated against a matrix of clinical and subclinical signs (**Supplementary Table 1**). A total score of 20 was considered a diagnosis of toxicosis, however, the attending veterinarian had the freedom to use their clinical discretion to diagnose toxicosis using fewer variables. Diagnosis of an individual dog with paralysis occurs when the dog reaches a total score of 20 across all 10 variables, or when the attending or consulting veterinarian uses their discretion to make a diagnosis based on one or more serious clinical signs. Once a dog was diagnosed with paralysis, the dog was removed from the study for clinical intervention.

### 2.5 ELISA Analysis of Sera From Trials 1 and 2

Sera from dogs at days 0, 14, 28, 42 and 50 from Trial 1 and at days 0, were screened in the HT ELISA as described above with the following changes. Following the blocking step, serial dilutions starting at 1:50 in BB of sera collected from each treatment group was dispensed across the plates and incubated for 1hr at room temperature (RT) on a platform shaker (Ratek Instruments, Australia). The negative control wells were coated with HT and probed with day 0 sera pooled from all dogs in experiment #1. Similarly, the positive control wells coating with HTs and tested with commercial Summerland purified anti-tick serum (TAS).

To determine the whole IgG and IgG1:IgG2 ratios from trial 2, the sera were screened using the HT ELISA with the following amendments. Sera were tested in triplicate, altering only the secondary antibody utilized each time. The secondary antibodies were: HRP conjugated- Sheep Anti-Dog IgG (abcam^®^, Australia); HRP conjugated- Sheep Anti-Dog IgG2 (Bethyl Laboratories, USA); and HRP conjugated- Goat Anti-Dog IgG1 (Bethyl Laboratories, USA), each diluted at 1: 10, 000 in BB.

The final titers for the ELISAs were determined as the reciprocal of the dilution that reached the background absorbance of the negative control.

### 2.6 Statistical Analyses

Data was transformed using the natural log (Ln2) to normalize before analysis using statistical software. All analyses were performed using GraphPad Prism software v6.0 (http://www.graphpad.com/scientific-software/prism/). Two-way ANOVA with a Tukey’s multiple comparisons post-test determined the variation between batches of TAS.

## 3. Results

### 3.1 Identification of HTs From the *Ixodes holocyclus* Transcriptome

Using the HT1 sequence (AAV34602) identified in the 1990s, our study identified a further 18 HT homologues (HT2-HT19) from the *I. holocyclus* internal organ transcriptome data11. Cocktail vaccine discovery described here screened all HT peptides except HT13 due to poor synthesis (data not shown).

### 3.2 Holocyclotoxin Immunogenicity Using Commercial Sera

**Figure 1:**
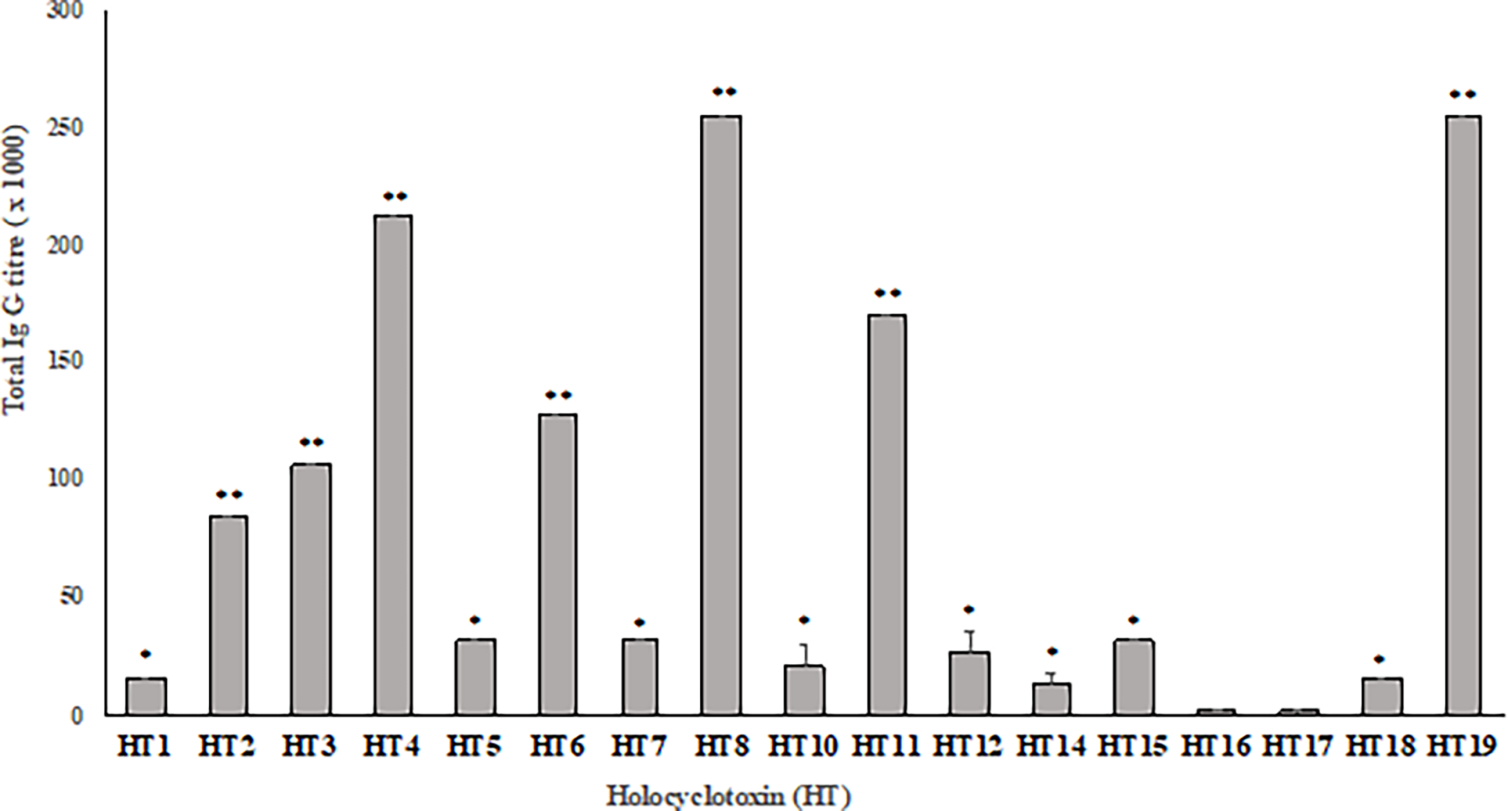
Average IgG titer against synthetic holocyclotoxins present in three different production batches of commercial tick anti-serum (Summerland). Results displayed as Mean± SD of end point titer (n = 3). The end point titer was determined as the reciprocal of the dilution that reached the background absorbance (OD λ = 450nm). Significance was determined by two-way ANOVA with a Tukey’s multiple comparisons post-test. ** (p < 0.05), * (p < 0.0001).

Production of different batches of commercial tick antiserum (TAS) obtained in 2014, 2015 and 2016 were used to determine the antibody titer against each synthetic holocyclotoxin. In ELISAs, these sera displayed a similar anti-HT IgG recognition pattern, as presented in **Figure 1**. The most significant anti-HT IgG titers were obtained for HT2, HT3, HT4, HT6, HT8, HT11 and HT19 when compared to all other toxins (p <0.05) with IgG titers ranging from 1: 80,000 to 1: 256,000. The rest of the HTs had significantly higher titers ranging from 1: 16,000 to 1: 32,000 when compared to HT16 and HT17 (p <0.0001). The lowest IgG titers were observed for HT16 and HT17 with 1: 2,000 and 1: 1,500, respectively.

### 3.3 Dog Immunization Trials

#### 3.3.1 Trial 1 – Folded *vs* Unfolded Holocyclotoxin Cocktail Vaccine (5 HTs) Tick Challenge in Dogs

Trial 1 aimed to investigate the safety of dog vaccination using folded *versus* unfolded HTs mixed in a cocktail of: HT1, HT2, HT3, HT4 and HT12 using a single dog for each group. The dog immunization was conducted on Days 0, 14 and 28 with only one dog per treatment including folded HTs (62469), unfolded HTs (63458) and a placebo (70084). The analysis of the paralysis tick symptoms in the folded HT dog (62469) showed only mild signs of tick toxicosis with tick removal or treatment not required (**Table 1**). This dog (62469) had an adult *I. holocyclus* tick feeding throughout the whole challenge. Similar results were observed for the placebo however, on final observations (144 hours post tick attachment), no ticks were found on this dog. However, the dog immunized with the unfolded HTs (63458) showed symptoms of tick paralysis, and the tick was removed at 84 hours post tick attachment (**Table 2**).

**Table 1:** IgG titers from dog Trial 1 with individual dogs immunized with Folded and Unfolded HT formulations consisting of HT1, HT2, HT3, HT4 and HT12.

HTs	1^st^ Dose	2^nd^ Dose	3^rd^ Dose	Tick Infestation	Post Tick Infestation
	Day 0	Day 14	Day 28	Day 42	Day 50
**Folded**					
HT1	0	3200	6400	800	400
HT2	0	200	6400	3200	51200
HT3	0	0	200	1600	12800
HT4	0	0	800	12800	51200
HT12	0	0	400	3200	51200
**Unfolded**					
HT1	0	200	1600	800	800
HT2	0	50	800	3200	25600
HT3	0	0	1600	1600	25600
HT4	0	0	400	800	12800
HT12	0	0	800	3200	25600

Immunization schedule and tick infestation days are described.

**Table 2:** Trial 1 clinical data for cocktail vaccine HT1, HT2, HT3, HT4, HT12 as folded *vs* unfolded immunizations.

Treatment group	Clinical observations post tick attachment (PTA)		Score^a^	Treatment
	Hours PTA	Observations		
Group 1 #70084 Placebo	102	slightly unwell	8/40	–
	105	Mild signs of tick toxicosis	5/40	–
	120	Mild signs of tick toxicosis	3/40	–
	123	Mild signs of tick toxicosis	3/40	–
	144	(no attached tick found)	3/40	–
Group 2 #62469 Folded HTs	102	slightly unwell	8/40	–
	105	Mild signs of tick toxicosis	5/40	–
	120	Mild signs of tick toxicosis	4/40	–
	123	Mild signs of tick toxicosis	1/40	–
Group 3 #63458 Unfolded HTs	81	Unwell, sore feet, did not jump, reduced gag reflex, bright and alert	6/40	–
	84	Quiet, did not jump, stumbled, appetite maintained	11/40	Tick removed
	87	Similar to 84hrs	8/40	–
	90	Similar to 84hrs	9/40	–
	93	Improvement noted	5/40	–
	96	Almost normal	0/40	–
	102	Sore on back, reluctant to walk	NA	–

a Maximum score is 40 which describes severe paralysis and poor prognosis. Total score for developing toxicity which required withdrawal from the study was >12 (or <12 at the veterinarian’s discretion); NA, not applicable.

Specific HT immunoglobulin induction was observed in the dogs receiving the folded and unfolded formulations of the pilot vaccine (**Table 1**). In dogs immunized with folded HTs and unfolded HTs formulations, the IgG titers against HT1 and HT2 increased after the first immunization, but no IgG titer was detected for HT3, HT4, HT12. A boost of the IgG titer occurred after the second and third immunization for all HTs in both formulations, at 28 and 42 days, see **Table 1**. Following tick infestation (at 50 days) the IgG titers were boosted for all HTs with a maximum increase for HT2, HT3, HT4 and HT12 in the folded HT (62469) and the unfolded HT (63458) immunized dogs. At this time, the anti-HT1 IgGs appeared to not change in both dogs immunized with folded and unfolded formulations. Two-way ANOVA with Tukey’s post-test identified a significant increase in the end point titers for all HTs compared to day 14 titer (p<0.001) excluding HT1. This trial had only a single dog per group, the placebo group tick challenge was not successful. However, it can be concluded that a folded HT formulation protected the dog from tick paralysis after an active challenging with an adult *I. holocyclus* tick. The unfolded peptide formulation induced non-protective antibodies as paralysis symptoms were observed after tick challenge. The tick paralysis symptoms in this dog (63458) decreased following removal of the *I. holocyclus* tick.

#### 3.3.2 Trial 2 – Cocktail Vaccine (8 HTs) Tick Challenge in Dogs

Trial 2 included a combination of eight HTs with four HTs selected due to high immunogenic recognition by polyclonal dog antisera, HT2, HT4, HT8 and HT11, see **Figure 1**. The subsequent four HTs were selected to guarantee that sequence variation of the entire HT protein family was included within the cocktail, HT1, HT12, HT14 and HT17. Note that HT19 was also highly immunogenic, yet its synthetic production was unsuccessful, and thus HT19 was not added to the cocktail. Immunizations and placebos (4 dogs per group) were delivered on days 0, 28 and 49 followed by challenge with one unfed adult female *I. holocyclus* tick at day 62. **Table 3** summarizes the clinical observations for Trial 2.

**Table 3:** Trial 2 clinical data for cocktail vaccine HT1, HT2, HT4, HT8, HT11, HT12, HT14 and HT17 as the cocktail immunization compared to the placebo group.

Treatment group	Dog ID	Clinical observations post tick attachment (PTA)		Score^b^	Treatment
		Hours PTA^a^	Observations		
Placebo Group I	62458	117	Overall clinical toxicity 1^st^ quartile Overall disease judgment 1^st^ quartile	2/40	
		153	Walks in circles with difficulty; climbs stairs with difficulty Overall toxicity in the 1^st^ quartile; Paralysis recorded In the first quartile, NMJ^c^ test showed mild weakness ataxia. Overall toxicity judgment in the first quartile. Hind leg weakness (wobbly when walking/standing)	7/40	
		156	Signs worsened drastically; Scored 3 out of 4 on 7 of the 12 tests	25/40	Tick removed, treated with TAS, fully recovered
	63061	168	Mild decrease in appetite, no other signs of toxicosis, ticks still attached	0	
	66324	144	Lost engorged tick during assessment	0	
		150	Climbed stairs with difficulty, mild weakness/ataxia in NMJ test Slight signs of hind leg weakness	3/40	
		156	Climbed all stairs but did not jump down Slight signs of hind leg weakness	1/40	
		168	Climbed stairs with difficulty, NMJ^c^ test showed mild weakness/ataxia toxicity score in first quartile, paralysis score in first quartile. Overall judgment of toxicity score in first quartile. Ataxic and weak hind legs; Episode of labored breathing.	6/40	Tick removed, fully recovered without treatment
	99768	96	Tick could not be located, did not develop signs of toxicosis	0	
Treatment Group II	34295 64799 68888	168	Engorged tick detached before final health check, no signs of toxicosis	0	
	55891		Still had engorged tick at health check; dog quiet and low body temperature otherwise no signs of toxicosis	0	

a Time points where all observations were zero are not shown, except the last day of observations.

b Maximum total score is 41 which describes severe paralysis and poor prognosis. Total score for developing toxicity which required withdrawal from the study was >12 (or <12 at the veterinarian’s discretion).

c NMJ, neuromuscular junction.

Administration of the vaccines was well tolerated. After the first vaccination on day 0, approximately 3 hours after the injection, a mild swelling at the injection site was observed in one of four placebo dogs (63061) but did not appear to cause pain and quickly resolved. Two of the four dogs (62458 and 66324) in the placebo group developed clinical signs of tick toxicosis on day 69 at approximately 150 hrs post tick attachment (PTA). At 156 hrs PTA, one control dog (62458) required intervention and was treated with tick anti-serum (TAS) resulting in a full recovery. For the other placebo dog (66324) that displayed signs of toxicosis, removal of the tick was sufficient to reverse the clinical signs and no further treatment was necessary. At 96 hrs PTA, the tick attached to dog 99768 could not be located and this dog did not develop signs of toxicosis. Dog 63061 in the placebo group did not develop signs of toxicosis despite the tick remaining attached until the final health check on day 70, although a mild decrease in appetite was observed on days 69 and 70. **Table 2** describes the clinical observations that were made for the placebo treated animals (Group 1) from day 67 where there was a clinical observation greater than 0. **Table 2** also summarizes the clinical observations that were made on the last day of trial 2 for the immunized animals (Group 2), no signs of toxicosis were identified at all previous health checks for the immunized group. Ticks stayed attached to all of the dogs in the immunized group (Group 2) until the final day (Day 70-final day of assessment, 168 hrs post tick attachment). None of the immunized dogs developed any signs of toxicosis.

**Figure 2:**
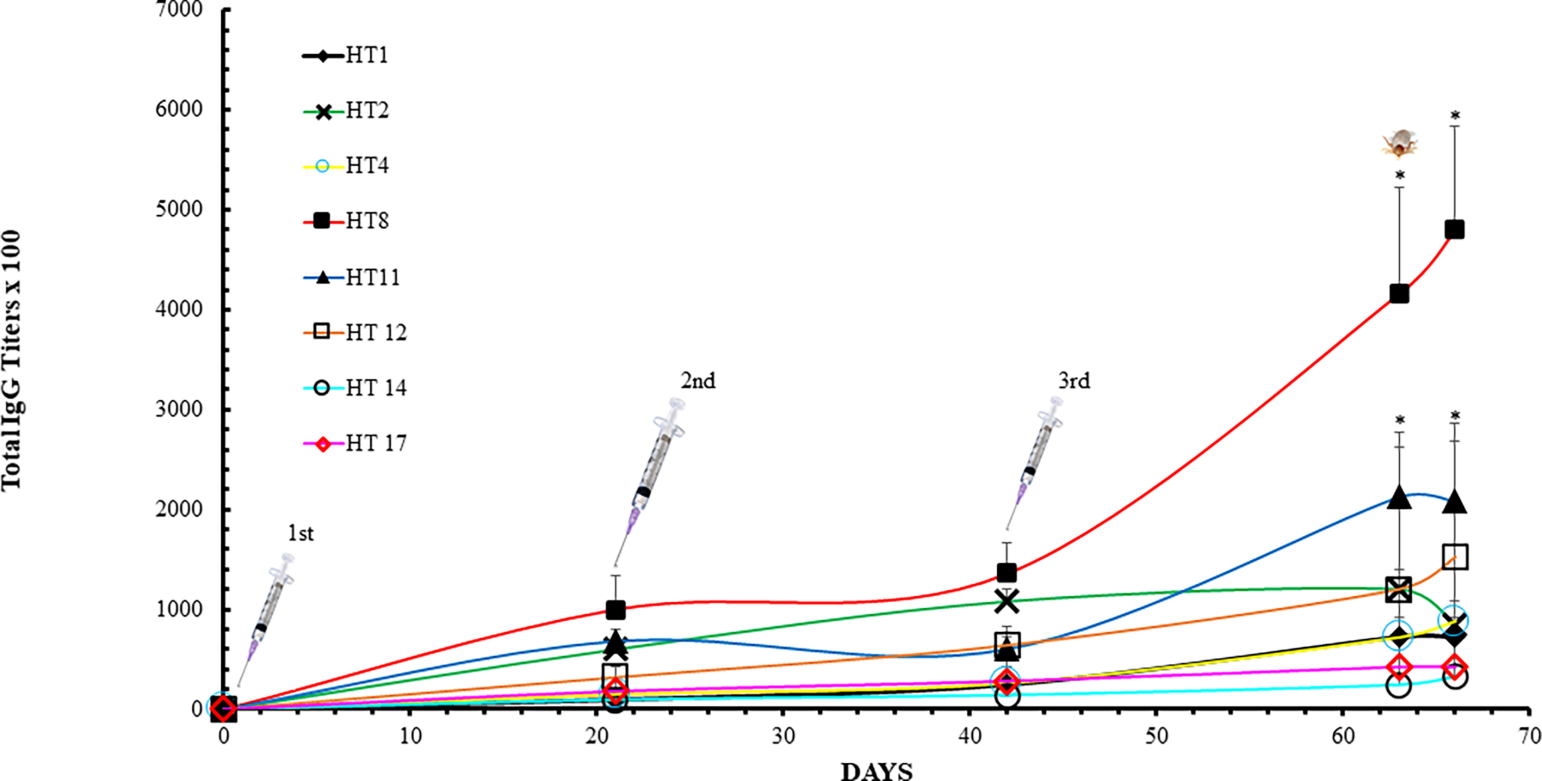
ELISA analysis with dog sera collected in Trial 2. Dog anti-holocyclotoxins IgG titer average displayed as mean ± SD after immunization with HT cocktail: HT1, HT2, HT4, HT8, HT11, HT12, HT14 and HT17. The titers were determined as the reciprocal of the last dilution that gave a positive signal in comparison to the background absorbance from the placebo dog IgG values. Arrows represent dog immunization dates (Days 0, 28 and 49). The tick identifies the date dogs were challenged with *I. holocyclus* unfed adult female ticks. Data were analyzed by Two-Way ANOVA with Tukey’s post-test (*p < 0.05).

Trial 2 observed an induction of HT specific IgGs in the dogs receiving the HT cocktail vaccine (**Figure 2**). The IgG titer induced against the HTs included in the cocktail increased during the course of the trial, except for HT2, whose IgG titer decreased between days 63 and 66. Spikes in IgG titers were observed after each inoculation. The most antigenic toxins were HT8 and HT11 with average titers between 232,000 and 480,000 at day 63. Two-Way ANOVA with Tukey’s post-test determined HT8 and HT11 to be significantly increased at days 63 and 66 compared to all other HTs (p<0.05). At day 66, the least antigenic holocyclotoxins were HT14 and HT17 with average titers of 32,000 and 42,000, respectively. Analysis of anti-HT IgG titers for individual dogs are shown in **Supplementary Figure 1**. IgG ELISAs of the dog serum samples analyzed at day 14 showed that IgG2 was the principal IgG subclass developed against all HTs under experimentation (**Table 4**). The IgG1:IgG2 ratios ranged from 1:1 to 1:64, however HT vaccinated dogs 34295 and 68888 had identical IgG1:IgG2 ratios for HT2 (1:1) and HT4 (1:1).

**Table 4:** The IgG1:IgG2 ratios against each holocyclotoxin within the cocktail vaccination at 14 days post immunization in trial 2.

Dog ID	IgG1:IgG2 titer ratio							
	HT1	HT2	HT4	HT8	HT11	HT12	HT14	HT17
34295	1:4	1:4	1:1	1:2	1:4	1:4	1:8	1:2
55891	1:16	1:16	1:16	1:16	1:64	1:32	1:8	1:4
64799	1:8	1:4	1:4	1:8	1:8	1:4	1:16	1:4
68888	1:2	1:1	1:1	1:4	1:4	1:2	1:16	1:4

## 4. Discussion

The primary host of *I. holocyclus* are Australian native marsupials such as bandicoots, whereas livestock, companion animals and humans are considered as secondary or incidental hosts. The adult female *I. holocyclus* produces the most harmful form of tick paralysis compared to its counterparts *Dermacentor andersoni* and *Dermacentor variabilis* in North America and *Rhipicephalus evertsi* in Africa (reviewed by 4, 5, 43). The severity of this toxicosis is observed principally in pets and domestic animals but there have been reports of fatal pediatric/pediatric cases (14, 26, 44–49). The clinical symptoms of paralysis ticks are loss of appetite, voice alteration and loss of limb coordination. Also, there is an ascending flaccid paralysis, excessive salivation, asymmetric pupillary dilation and respiratory distress, reviewed by (26).

Purification of neurotoxic components present in the paralysis tick’s saliva were elusive since the first report by Thurn et al. in 1992 (8) and a proteomics study undertaken in 2008 failed to identify holocyclotoxins (50). Up to twelve years ago, the resources of tick protein databases were limited and thus it was a bioinformatics hurdle to identify tick proteins using *de novo* sequencing and mass spectrometry. Currently, the situation is quite different with approximately 375 *I. holocyclus* proteins reported in NCBI (txid65647[Organism:noexp]), 149,746 proteins from *Ixodes scapularis*, and the *I. holocyclus* database deposited into Genbank (transcriptome shotgun assembly GIBQ00000000) associated with the manuscript originally describing the HT family published in 2018 (10). Holocyclotoxins are described as a ~ 5kDa peptide that in SDS-PAGE migrate at 40 – 80kDa (8, 40). It is not known if these large HTs from extracted gel fragments are produced only by HTs or whether HT carrier proteins are involved. This phenomenon has previously been described for *I. holocyclus* with ‘anti-toxin’ monoclonal antibodies recognizing 100-200 kDa proteins through western blot analysis (51). Similar inconsistencies have been identified for other paralysis causing tick species with different size toxic fractions found in *R. evertsi evertsi* (80-100kDa) and *R. evertsi evertis* monoclonal antibodies showed cross reactivity with *A. walkerae* toxins of different sizes (5, 52, 53). Transcriptomic studies on these other tick species to identify the coding regions of the corresponding toxins are yet to be reported. With the advent of improved tick genome sequencing combining improved long and short read technologies (54, 55), it may be feasible to sequence genomes which will assist to identify homologues of toxins in more tick species. The genome sequence of a paralyzing tick species is yet to be reported. Toxin or venom research reviews do not always mention ticks most likely due to the fact that only toxins for *I. holocyclus* have been recently characterized (10). Nonetheless, this study reports the development of an anti-paralysis tick vaccine based on a cocktail of neurotoxins produced in the venom of the adult female *I. holocyclus* tick.

Recently, toxin-related sequence descriptions were identified within the *I. holocyclus* dataset. Data showed that holocyclotoxins belong to a multigene family of a highly conserved inhibitor cysteine knot (ICK) motif (10, 56). These cystine-rich peptides are present in the venoms of scorpions and spiders and ICK motif conferred remarkable stability to these peptides (57). Following the discovery in *I. holocyclus* of a large holocyclotoxin (HT) family of ~19 peptides (10), this report confirms the identification of HTs included in a vaccine cocktail able to protect dogs from tick challenge. Our study demonstrates for the first time that folded HTs (cystine-rich peptide) induced a strong and protective immune response compared to unfolded holocyclotoxins. These toxins are cystine-rich peptides with disulphide bridges that provide conformational rigidity to the molecule, extreme stability to degradation by heat or enzymes (40, 41, 57). There is a previous report of anti-toxin peptide vaccination obtained using a linear epitope of the *Loxosceles intermedia* (recluse spider) dermonecrotic protein isoform (LiD1) showing a modest level of protection (58). However, a continuous B-cell epitope of 27 amino acid related to a fragment of the Smase D protein induced 75% protection *in vivo* for lethal doses of *L. intermedia* venom (59, 60).

The administration of an anti-paralysis tick serum (TAS) has demonstrated neutralizing potency after envenomation with the paralysis tick neurotoxins. It also has many drawbacks, including inhumane production (through dog hyper-immunization), limited window of efficacy (needing to be administered in the early stages of paralysis), non-standardized dosages, side effects associated with serum sickness, and varying potencies between batches and manufacturers (reviewed by 26). The paralysis caused by holocyclotoxins appears to be pre-synaptic (25), hence, the anti-tick serum (TAS) used in Australia is ineffective once the paralysis has progressed. Acaricides have been the most dominant form of preventative treatment. However, the development of tick resistance to chemicals and evidence of adverse reactions to certain chemicals for some pets demonstrate the need for a safe preventative such as a vaccine. In an attempt to remediate these circumstances for other anti-venoms, studies (20, 61–63) have used new biotechnological tools to improve the efficacy, safety, and cost-effective management of antivenin production, such as immunization with synthetic peptide epitopes, recombinant toxins (or toxoids), or DNA. These methodologies reveal the potential for producing antivenins with high therapeutic antibody titer and broad neutralizing capacity (20, 61). Also, these approaches avoid the use of venom in the production process, thus preventing the difficulties related with animal captivity and venom collection (20, 61–64). This study describes for the first time the development of a neurotoxin cocktail vaccine demonstrating dog protection from paralysis induced by female adult *I. holocyclus* ticks, which could also enable the production of safe anti-paralysis antivenins. Further larger trials would be necessary to confirm the protective efficacy of the vaccine described by this study using, for example, different breeds of dogs and a challenge with a higher number of ticks.

The high titers of anti-holocyclotoxin IgGs induced after immunizing dogs with the HT cocktail vaccine neutralized the symptoms of the paralysis tick. In these experiments, the IgG subclass predominantly stimulated was canine IgG2 (or IgG- B and C, as reported by Bergeron et. al., 2014 (65). Dog IgGs have been divided into four subclasses, A-D (66), and subsequently Bergeron et al. (65) showed that commercial anti-canine IgG1 recognized the dog IgG subclasses A and D while anti-canine IgG2 reacts with subclasses B and C. Based on the effector function, canine IgG subclasses are analogous to their human IgG counterparts. Canine immunoglobulins B and C are similar to human IgG1 and IgG3, but A and D IgGs are most similar to human IgG2 and IgG4 (65). These classes are known to be stimulated by soluble proteins, corresponding with our vaccine composition, and are potent triggers of effector mechanisms due to strong FcγR engagement in anti-toxin activity (67, 68). Canine IgG A and D are associated with more subtle responses caused by a weaker FcγR engagement (68, 69). In addition, canine IgG2 has previously correlated to the successful protection against diseases and parasites in dogs. For example, high IgG2 titers in dogs immunized with a cocktail peptide-based vaccine has been associated with protection against leishmaniasis (70). The evidence suggested that a preferential induction of the IgG2 subclass in dogs is correlated with a highly protective immune response. In this study, the immunization with HTs in an oil-based adjuvant developed hyper immunity in dogs without inducing symptoms of paralysis. Stone et al. reported different results, as they detected paralysis symptoms in partially immunized dogs (71, 72). These previous studies in optimizing the production of anti-tick IgGs in dogs and rabbits reported a very slow development and affinity maturity of protective immunity caused by using toxin preparations of questionable purity, and an inoculation regime of up to two-years to develop sufficient protection (72). Thus, in the pursuit of a tick-paralysis vaccine, synthetic or recombinant HTs offer a safe and cost-effective vaccine delivery method.

In conclusion, we report the successful immunization of dogs using a synthetic peptide toxin cocktail derived from a family of holocyclotoxins identified in the Australian paralysis tick *I. holocyclus*. Future research should focus on recombinant production of the HT peptides, determining optimal dosages, and vaccine longevity studies to ensure adoption of the vaccine to protect companion pets from paralysis.

## Data Availability Statement

The datasets presented in this study can be found in online repositories. The names of the repository/repositories and accession number(s) can be found below: https://www.ncbi.nlm.nih.gov/genbank/, GIBQ01000000.1.

## Ethics Statement

The animal study was reviewed and approved by Elanco Animal Health.

## Author Contributions

MR-V and AT (equal contributions and coordination of the research) conceived the project with support from KA, AK, and MB (project initiation and research management). Dog trial planning undertaken by AK, followed by CE and KV with the above acknowledged trial team to execute the trials. ELISA screening and the analysis of dog immune responses undertaken by SM and MR-V. The Honours thesis of SM contributed methods and results to this manuscript. Manuscript was equally prepared by MR-V and AT. Bioinformatics analyses (research component coordinated by MIB) and assistance towards the submission of the TSA to the Genbank database undertaken by MB and PM and coordinated by AT. Vaccine doses were prepared by MR-V. All authors contributed to the article and approved the submitted version.

## Funding

This research was supported by the Australian Research Council Linkage grant with Elanco LP120200836.

## Conflict of Interest

Author KA was employed by company Paul Dick & Associates Ltd. Author AGK was employed by company Virbac Australia Pty Ltd. Authors CE and KV were employed by Elanco Animal Health.

The remaining authors declare that the research was conducted in the absence of any commercial or financial relationships that could be construed as a potential conflict of interest.

## Publisher’s Note

All claims expressed in this article are solely those of the authors and do not necessarily represent those of their affiliated organizations, or those of the publisher, the editors and the reviewers. Any product that may be evaluated in this article, or claim that may be made by its manufacturer, is not guaranteed or endorsed by the publisher.

## Supplementary Material

Supplementary Table 1 Clinical Diagnosis Matrix for Tick Paralysis in Dogs. VAS, Visual Analog Scale.

Supplementary Figure 1 Dog anti-holocyclotoxin IgG titers of the animals immunized in Trial 2. Titers against each holocyclotoxins per dog are shown. The average SD titer of three replicates per time point of each dog serum are represented.
