# Supplementary material for: Immunomic Investigation of Holocyclotoxins to Produce the First Protective Anti-Venom Vaccine Against the Australian Paralysis Tick, Ixodes holocyclus
Source: Front Immunol. 2021 Oct 4;12:744795. doi: 10.3389/fimmu.2021.744795 (PMC8522651; doi:10.3389/fimmu.2021.744795)
Supplement: Supplementary Table 1 — Clinical Diagnosis Matrix for Tick Paralysis in Dogs. VAS, Visual Analog Scale. [file DataSheet_1.pdf]

Supplementary Table 1. Clinical Diagnosis Matrix for Tick Paralysis in Dogs.

Legend: VAS=Visual Analog Scale

**Test number**	**Test**	**Description**	**Scoring**	**Score**	**Paralysis developing at**
1	Bark test	Test of changes in vocalisation	1 = no change in vocalisation<br>2 = possible minor change in vocalisation<br>3 = change in sound of vocalisation<br>4 = unable to bark	/4	Score = 3
2	Eat test	Test acceptance of treat/food	1 = smells and eats treat<br>2 = smells and shows interest in treat but doesn't eat treat<br>3 = smells disinterestedly and does not eat treat<br>4 = shows no interest in treat	/4	Score = 3
3	Jump test	Response to trained request to jump	1 = jumps with vigour<br>2 = jumps with encouragement<br>3 = attempts to jump with encouragement but jump unsuccessful<br>4 = refuses to jump	/4	Score = 3
4	Stair climb test	Measure of number of stairs climbed (/4) and jump from top	1 = climbs all stairs and jumps off end<br>2 = climbs all stairs but no jump<br>3 = climbs <3 stairs<br>4 = does not attempt to climb	/4	Score = 2
5	Overall clinical toxicity VAS	Visual analog scale where clinician is asked to rate on a line between 0 and 10cm the severity of clinical toxicity	0 = VAS of 0<br>1 = <25 (1st quartile)<br>2 = 25–50 (2nd quartile)<br>3 = 50–75 (3rd quartile)<br>4 = >75 (4th quartile)	/4	Score = 2

6	Paralysis VAS	Visual analog scale where clinician is asked to rate on a line between 0 and 10cm the severity of paralysis	0 = VAS of 0<br><br>1 = <25 (1st quartile)<br><br>2 = 25–50 (2nd quartile)<br><br>3 = 50–75 (3rd quartile)<br><br>4 = >75 (4th quartile)	/4	Score = 2
7	Respiratory distress VAS	Visual analog scale where clinician is asked to rate on a line between 0 and 10cm the severity of respiratory distress	0 = VAS of 0<br><br>1 = <25 (1st quartile)<br><br>2 = 25–50 (2nd quartile)<br><br>3 = 50–75 (3rd quartile)<br><br>4 = >75 (4th quartile)	/4	Score = 2
8	Neuro-muscular junction test	Test of neuromuscular function based on movement	1 = good<br><br>2 = mild symptoms<br><br>3 = moderate symptoms<br><br>4 = severe symptoms	/4	Score = 2
9	Toe pinch test	Test of time to respond to moderate pressure applied to a rear limb toe	1 = immediate retraction<br><br>2 = slowed retraction response<br><br>3 = moderate delay in retraction<br><br>4 = no response to pressure	/4	Score = 2
10	Overall intuitive judgement	Measures clinician's response to all symptoms	1 = normal healthy dog<br><br>2 = dog showing abnormal signs<br><br>3 = dog showing early signs leading to paralysis<br><br>4 = Dog is definitely showing signs of paralysis and requires clinical intervention	/4	Score = 2

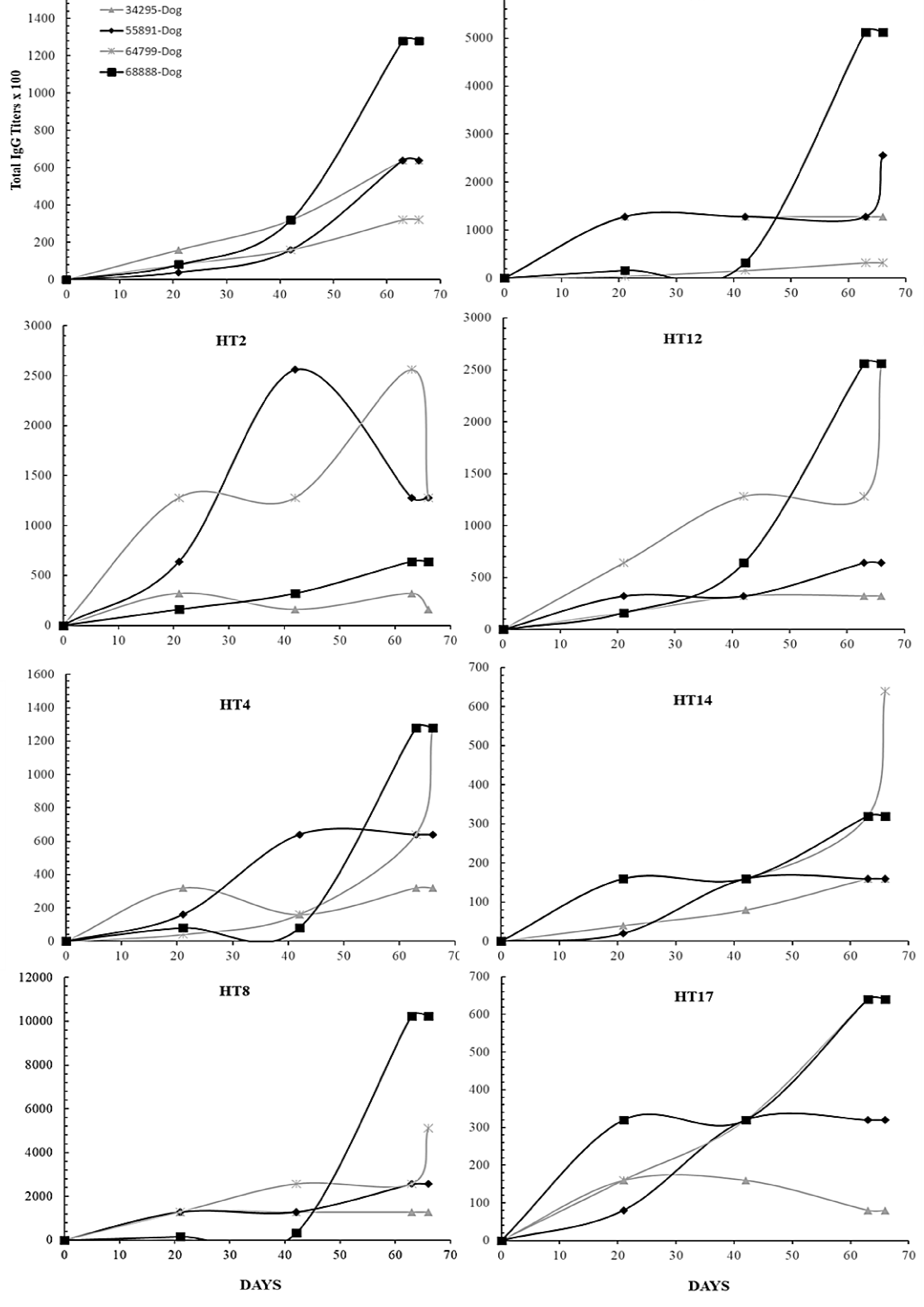
34295-Dog
55891-Dog
64799-Dog
68888-Dog
Total IgG Titers x 100
HT2
HT12
HT4
HT14
HT8
HT17
DAYS
DAYS
